# On the Temporal Stability of Analyte Recognition with an E-Nose Based on a Metal Oxide Sensor Array in Practical Applications

**DOI:** 10.3390/s18020550

**Published:** 2018-02-11

**Authors:** Ilia Kiselev, Victor Sysoev, Igor Kaikov, Ilona Koronczi, Ruslan Adil Akai Tegin, Jamila Smanalieva, Martin Sommer, Coskan Ilicali, Michael Hauptmannl

**Affiliations:** 1 Breitmeier Messtechnik GmbH, Englerstr. 27, 76275 Ettlingen, Germany; hauptmannl@breitmeier.de; 2 Laboratory of Sensors and Microsystems, Yuri Gagarin State Technical University of Saratov, 77 Polytechnicheskaya str., 410054 Saratov, Russia; 3 National University of Science and Technology MISiS, 4 Leninskiy pr., 119991 Moscow, Russia; 4 Institute of Microstructure Technology, Karlsruhe Institute of Technology, Hermann-von-Helmholtz-Platz 1, 76344 Eggenstein-Leopoldshafen, Germany; igor.kaikov@gmail.com (I.K.); martin.sommer@kit.edu (M.S.); 5 Science and Technology of Nanosystems, Karlsruhe Institute of Technology, Hermann-von-Helmholtz-Platz 1, 76344 Eggenstein-Leopoldshafen, Germany; ilona.koronczi@kit.edu; 6 Faculty of Engineering, Kyrgyz-Turkish Manas University, Mira Avenue 56, 720044 Bishkek, Kyrgyz Republic; ruslan.adil@manas.edu.kg (R.A.A.T.); jamila.smanalieva@gmail.com (J.S.); coskan.ilicali@gmail.com (C.I.)

**Keywords:** electronic nose, instability, long-term stability, ambient air, meat quality control, honey recognition, linear discriminant analysis

## Abstract

The paper deals with a functional instability of electronic nose (e-nose) units which significantly limits their real-life applications. Here we demonstrate how to approach this issue with example of an e-nose based on a metal oxide sensor array developed at the Karlsruhe Institute of Technology (Germany). We consider the instability of e-nose operation at different time scales ranging from minutes to many years. To test the e-nose we employ open-air and headspace sampling of analyte odors. The multivariate recognition algorithm to process the multisensor array signals is based on the linear discriminant analysis method. Accounting for the received results, we argue that the stability of device operation is mostly affected by accidental changes in the ambient air composition. To overcome instabilities, we introduce the add-training procedure which is found to successfully manage both the temporal changes of ambient and the drift of multisensor array properties, even long-term. The method can be easily implemented in practical applications of e-noses and improve prospects for device marketing.

## 1. Introduction

A sufficient time has gone since introduction of the electronic nose (or e-nose) concept, which harks to a pioneering paper by Persaud and Dodd [1]. In this concept, the e-nose is a device that consists of a multisensor array and multidimensional signal processing of the array signal by pattern recognition algorithms [2]. Since then, numerous research groups have tried out the concept, which has resulted in appearance of a number of such device units, also in a commercial market, based on various gas-sensor platforms [3,4].

The development of highly gas-sensitive materials and their proper adoption in transduction units have been combined with achievements in multivariate data analysis and computer engineering capabilities [4,5,6,7]. Nowadays, the available e-noses are ready to meet the needs of various industrial applications. However, the decisive goal, the intended market penetration, has not been reached yet [8], and as stated by many authors [4,9,10,11,12,13,14,15], real working applications of e-noses can hardly be found. The reasons for the problem are systematically discussed in [9].

In our opinion, based on many years’ experience and our review of the available literature, the major reason that hinders these devices from occupying a proper application niche is their instability during continuous ongoing use. The appearing instability has resulted in suspicion of the expert community that e-noses are not sufficiently reliable for practical use, especially in the long term. As a consequence, a number of research and e-nose production programs have been phased out. The “instability” of the devices mainly means losing the ability to recognize target odors over time following an initial training. Although the after-training tests of e-noses usually show a quite good recognition of target objects, this degrades frequently in following tests under long-term operation [16]. Sometimes, the e-noses, which have perfectly functioned at the primary tests, lose their ability to identify the odors rather quickly [12,17].

Other characteristics of e-noses such as sensitivity, selectivity, robustness, power consumption and cost are also under consideration in the community. The sensitivity and selectivity are very frequently considered as the major topics of research for many labs dealing with e-nose and/or gas sensors in general [3,4,18,19]. These issues are abundantly reported to be sufficient for many practical applications where a high discrimination of gases at low concentrations is not necessary [20,21]. Indeed, many lab investigations, the great majority of which are performed under rather short-term conditions, are proved to satisfy the requirements of end users. Thus, selectivity and sensitivity do not impede marketing of the e-nose. As for other parameters, e-noses are usually quite robust (see, e.g., [22]) and of lower cost when compared to that of other known gas-analysis methods [4,23]. Therefore, the instability of e-nose functional properties seems to be the key point to address when considering their use.

Still, there is one more related issue to mention. Each multisensor array is unique as the primary “receptor/transducer”. It cannot be replaced in the same e-nose unit without re-calibration (or re-training). Were the arrays reproducible like ordinary electronic components such as resistors, capacitors, FETs, etc., any cost required to calibrate an e-nose versus any possible environment would be worth paying because the pattern recognition would not need to build a new knowledge database. In this case, the generated calibration model patterns corresponding to gases or gas mixtures in the e-nose database could be applied to other multisensor arrays of that particular type. However, today’s multisensor arrays are rather individual, although the need to make them reproducible is frequently addressed [10,14,24,25]. So far, there are only few reports that have considered producing comparable sensors, but even these sensors are found to deviate in performance with time of e-nose exploitation [16], which again refers us to the temporal instability of the device operation. The possibility to transfer a calibration dataset created by one e-nose unit versus some chemically pure analytes to another e-nose unit has been tried out in [26,27]. However, the effectiveness of the procedure needs further validation for practical applications.

The problem of e-nose instability can be approached by means of signal processing. In particular, there exists an opinion that applying certain refinements to the multisensory data could allow one to extract the steady recognition features related to test gases/gas mixtures of interest which would not depend on instabilities of the sensor array signal [10,28,29]. However, there is an insoluble obstacle in this notion that we can anticipate: when a new unknown gas atmosphere appears the e-nose has to deal with this new information, which has been absent in the initial training pool. This means that there is no other way to predict the behavior of the multisensor array except for incorporating the appeared new information into the training pool. This is a good reason to seek managing unpredictable e-nose instabilities and not depend on data processing/feature extraction.

Many authors point out different factors as the main causes of e-noses’ instability, among which the temporal drift of the sensors themselves is most frequently considered to be the major one [9,12,17,20]. In fact, any type of the known sensors which compose e-noses is subject to drift [4,17] except possibly for a few examples [30]. Of course, various sensor types exhibit their own mechanisms responsible for the drift. Nonetheless, all the reported drifts have usually a systematic characteristic of temporal changes of sensor properties [16,17]. Therefore, contrary to unpredictable instabilities, the sensor drift can be approached by using suitable data processing methods. An example of successful sensor drift compensation under a synthetic air ambient for a period of up to twenty days of operation is given in [26]. However, the drift of physicochemical properties of sensors is mainly observed on the scale of months and longer. As a rule, the possible short-term drift is eliminated already at the sensor fabrication stage, e.g., by accelerated aging procedures [17].

Another source for e-nose operation instability is sometimes seen in poisoning of the sensors [11,13,19,20]. However, this problem usually appears when e-noses are exposed to certain gases which might create immobile reaction products. This effect depends greatly on the sensor type. To the best of the authors’ knowledge, thin-film metal-oxide chemiresistors are so far the most reliable sensor units versus many gases because they are rather stable gas-sensitive materials that work at high operation temperatures, which facilitates an easy removal of reaction products. In general, the poisoning could be avoided in many cases by proper adjustment of the gas sampling facility and procedures [20]. Therefore, poisoning is not among the factors which require urgent attention to have stable working e-noses.

The varying environmental conditions are also frequently mentioned as the source of instability of e-nose operation, especially if the devices have to operate in ambient air. This mostly concerns the air temperature [9,15,16] and humidity content deviations. Indeed, the temperature variations could be a significant drawback for e-noses based on multisensor arrays working under so-called “room temperature” conditions or being heated without implementation of built-in sensor temperature stabilization [17,25]. However, known ordinary electronics could compensate such variations if properly installed in the device. In contrast, the influence of humidity is a more general factor affecting multisensor arrays, as extensively debated [9,14,16]. Because the presence of water vapor might significantly change the chemistry of the interactions of other substances with the sensitive materials, e-noses can behave differently depending on the humidity level in the air. A possible way to overcome the impact of humidity is a calibration of e-noses to an application-specific range of humidity variations [15], although the straightforward way is still designing the multisensor array based on sensors whose signal does not depend on humidity so much [24]. Here we consider the variations of water vapor concentration as just one of the components of the ambient air composition that influence the e-nose performance.

In spite of the listed instability factors, we try to show here that the currently available e-noses can work stably even on the long-term scale. We approach this critical issue considering primarily the e-nose based on a chemiresistive metal-oxide multisensor array. However, we hope that the reported findings could be useful for e-noses based on other sensor types, too. We arrange the paper to address various time scales of e-nose operation, ranging from minutes-hours (short-term) to weeks-months (mid-term) and up to many years (long-term) to discuss them in the corresponding sections.

## 2. Instrumentation and Methods

The e-noses employed in this study are of the same portable type designed for practical applications, primarily known under the KAMINA abbreviation. Their construction is described in details elsewhere [23,31]. Briefly, it is based on a multisensor array chip, which carries 38 chemiresistive sensor elements based on SnO_2_/Pt thin films. The sensor elements are formed by segmentation of the film with a set of co-planar parallel Pt electrodes over Si/SiO_2_ substrate; therefore, hereafter they are called segments. The segment resistances are sequentially read out by e-nose electronics in cycles with the rate up to 1 Hz per the whole array. The set of segment resistances measured in one cycle is considered as a multidimensional data point of the e-nose. In order to enhance the functional dissimilarity of the segment properties over the array, a temperature gradient of approx. 10 °C/mm is spatially applied along the chip sensitive surface [32]. The temperature at the hottest edge of the chip is maintained by electronics with an accuracy of ±1 °С. In most experiments reported here, the maximum temperature has been set to 300 °С, except for the data related to the long-term study where the hot-end temperature was equal to 330 °C. At the beginning of measurements, no sensor chips have been “as-prepared” being already under the operation at elevated temperatures for several weeks at least. All the measurements have been performed in ambient air; the analyte probes have been delivered to the chip at the rate of about 1 L/min through a rather small, less than 3 cm^3^ of internal volume, stainless steel chamber equipped with an oil-less miniature diaphragm pump (model 3003VD, Gardner Denver Thomas, Wayne, PA, USA). The response time of e-nose itself is less than 5 s [23,33], but it extends up to 10 s depending on the odor delivery system [31] in all the measurements, except for the exposures of the e-nose to honey, where it increases even up to 30 s. The recovery time depends less on the surrounding equipment and amounts to approx. 60–100 s [33]. Special care has been addressed to operate the e-nose close to real-life applications. The honey and pieces of fruits to be analyzed by the e-nose have been furnished in Petri dishes, of approx. 150 mL volume. The amount of substances has varied from several mL to several tens of mL (honey, fruits) or a few grams (meat). The meat samples have been exposed in phials of approx. 0.5 L volume. After every exposure to an analyte, the e-noses has been exposed to ambient air for at least few minutes.

The measured segment resistances of the multisensor array are considered as signals to process. In order to reveal the appearances of the analyte odors the e-nose traces the median resistance value taken over the array segments. An exposure of *n*-type metal oxide sensors to organic analytes of reducing nature causes a decrease of resistance value (see e.g., [23]) when compared to that recorded in air. A sequence of the data points recorded prior the analyte introduction is considered as the sampling of ambient air. The data points observed around the point with the minimum median value at an analyte probe are considered to be the sampling of this probe. The selection of data point sequences for the samplings is done either automatically (in Section 3.3) or by the operator. The number of points in the samplings is in the range of 20–100.

To eliminate the signal dependence on analyte intensity (or concentration), the segment resistances are normalized by the median value. The procedure is considered as the most convenient one to that end and is widely used in our common practice [31,34], although there are other methods specifically developed for this task [35,36]. However, in the short-term study (Section 3.1), the pattern recognition algorithm uses the raw resistances as the data for the following processing. We have employed the Linear Discriminant Analysis (LDA) algorithms to process the samplings as sets of multidimensional data points (or, of array profiles from the viewpoint of value distribution over the array). The LDA method is one of the powerful techniques which reduces the data generated by the multisensor array to the space where the target analyte clusters are separated to the maximum degree [37]. However, we guess that the kind of pattern recognition algorithm, if properly optimized, does not have a significant effect to the problem of e-nose instabilities, because the latter ones are caused by the mode of multisensor array operation, which affects the algorithms evenly. However, LDA is possibly the best technique to visualize the analyte discrimination by the array. In all the cases, except for those specially indicated in Section 3.3, the recognition of multisensor e-nose data in the LDA space has been performed in the frames of a Voronoi diagram [38,39]. In this method, every test data point is classified to belong to the analyte class whose LDA center is located at the closest distance. The full data sampling recorded by the e-nose under exposure to an analyte is classified into one of the training classes when more than 50% of the sampling data points are classified into the corresponding class. To analyze the e-nose data further, we use here a so-called recognition score, which is defined as a ratio of number of the correctly recognized samplings to the total number of the sampling. The class boundaries are defined by Gaussian distribution taken at 0.95 confidence probability, which frames analyte-related ellipsoids in the LDA multidimensional space. At the discussion of Section 3.3, the E-nose data which appear outside all these analyte-related ellipsoids are qualified as “outliers”.

As the main tool to examine the stability of analyte recognition by the e-nose, we may analyze the function of recognition score *S*(*N*) in dependence on increasing number of e-nose data samplings *N* included into the training pool, as discussed in [34,36]. Indeed, the common way to reach a stable analyte recognition by LDA as well as by other pattern recognition algorithms goes via attempt to include all possible pattern variations related to target analytes into the training pool [4,9]. In this way, the *S*(*N*) function grows with the increasing *N*. A trainer has only to control the stabilization of the recognition level: when this level is stable, the training is complete. The two kinds of *S*(*N*) curves could be employed to observe the stabilization. The first one is Test-One-Measurement *S_TOM_*(*N*) function, which represents the leave-one-out (LOO) averaged recognition score [40] in dependence on the increasing *N* [34]. The second one is Test-Rest-Measurements *S_TRM_*(*N*) function, which shows the recognition of the e-nose data of a sampling series measured later than that included into training pool. Thus, *S_TOM_*(*N*) indicates how stable is the current e-nose recognition of the analytes, whereas the *S_TRM_*(*N*) characterizes the capability of the LDA model to recognize the e-nose data following the training procedure. The two *S*(*N*) functions characterize different aspects of the recognition stabilization (cf. Section 3.2).

To overcome the inherent sensor array drift and the air variations of different kinds we consider here a so-called add-training procedure. By means of add-training, the model database of an e-nose is updated in an ongoing fashion involving new current sampling data. We prefer using this term rather than “recalibration” in order to underscore the regularity of the procedure and its direct relation to the initial training. From the viewpoint of the temporal instabilities, the add-training procedure consists of periodical continuous introduction of the analyte probes of interest to the e-nose and adding these data into the training pool of the LDA model. At the same time, the add-training procedure allows one to discard obsolete data stored earlier in the training pool of the LDA model.

## 3. Experiments, Results, and Discussion

### 3.1. Short-Term Instabilities

Primarily, we consider e-nose operation immediately following its training. The training in this case consists of single e-nose exposures to each target analyte added to ambient air as a background. Here we call the LDA models based on such a database elementary ones. While the conventional e-nose training procedure includes numerous measurements of the target analytes in order to build the training pool which encompasses possibly more variations, the idea to introduce the elementary models here consists of tracking the transients which occur in the course of a short-time period after the acquisition of the training pool data. In particular, in this study each training pool has been collected within a quarter of an hour. Then, the following e-nose test exposures have been carried out at increasing time intervals.

**Figure 1:**
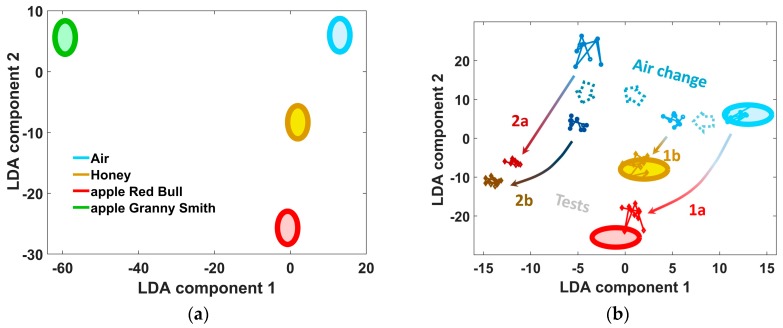
The e-nose data tested at the short-term scale versus elementary LDA model. The ellipses indicate the positions of training class data obtained at elementary model mode. (**a**) The position of analyte-related ellipses in the LDA elementary model; (**b**) two rounds of analyte recognition by the E-nose. “a” and “b” designate the test e-nose data transitions at the exposures to apple of first sort and honey, respectively. The e-nose data measured at round 1 (1a and 1b) have been taken about 15 min after the collection of training sampling and within 15 min one after another. Then, after a pause of 40 min, the e-nose data have been collected at round 2 (2a and 2b).

**Figure 2:**
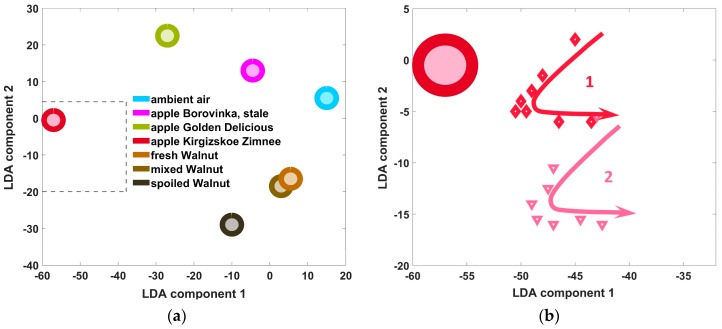
(**a**) General view of the class ellipses in LDA space illustrating the e-nose model built to recognize apples of different kinds (“Borovinka”, “Golden Delicious” and “Kirgizskoe Zimnee”) and walnuts (“Ak-Terek”) of different freshness; (**b**) Enlarged area framed in the plot (a) to draw e-nose data trajectories at the LDA space during exposures to Kirgizskoe Zimnee apple at the first (1) and second (2) rounds following LDA model building. The e-nose data of 2nd round are more than two times farther away from the training class position than these of 1st round.

The examples of the elementary training and test e-nose data projected into 2-component LDA coordinate space are illustrated in Figure 1 and Figure 2. All the samples were exposed in Petri dishes; the fruits have been previously cut to pieces (with one exception below), and the nuts have been shelled. The analyte odor samples were taken directly from the food containers by slightly lifting the lid in the framework of non-static headspace measurement [11] to be delivered to the e-nose. As the analytes for the first elementary model (Figure 1), we utilized the odors yielded by apples of two sorts, “Red Bull”, “Granny Smith”, and a honey (trademark “Wild honey”). The Granny Smith apple was measured as a complete fruit: its odor was measured by an e-nose in close proximity to the apple without any storage container. Immediately after the primary exposure of e-nose to the analytes and building the elementary LDA model, we have conducted the first recognition tests. At this first round of tests, the e-nose was exposed to three analytes within 15 min in sequence. The similar second round of analyte exposures were carried out after a pause of 40 min. The measurements of the second example (Figure 2) were performed in a similar manner.

In Figure 1, the ellipses frame the e-nose training datasets while the points indicate the e-nose data related to test analytes to be recognized as listed in the legend. The perfect separation of the LDA projections of training data clusters (Figure 1a) is rather typical for elementary LDA models. The plot of Figure 1b shows how the LDA projections of the e-nose data go out of the position related to ambient air recorded just before the e-nose exposure to the positions related to test analyte probes. The numbered arrows indicate the transitions of the e-nose signals from the air-related clusters to the corresponding analyte-related clusters in the LDA space. We can clearly see that the e-nose signals recorded both in air and in the analyte shift away from the primary training data clusters–classes of the LDA model.

The shifts become larger with increasing time after the training. The two exposures of e-nose at the first round (1a and 1b) to test analytes just after the training yield signals which match quite well the training data: the points are placed close to or within the training ellipsoids. The positions of the e-nose signals in the LDA space at the second test round (2a and 2b) drift from the corresponding training ellipses far away when compared to data observed at first round of exposures. It is worth noting that the results given in Figure 1 are quite typical. The similar trend, when the recognition of the analyte probes by e-nose at the second test round is worse, has been observed, more or less expressed, with all other elementary models, too. An additional example is shown in Figure 2. Obviously, these results cannot be explained by the sensor drift, which occurs at larger time scales as discussed in [16,17]. Neither can sensor poisoning be the reason for this e-nose signal shift because in that case even the first-round tests would result in deviation of e-nose signals far from the training ones. Moreover, these changes of the device response cannot be ascribed neither to variations of humidity content nor to temperature variations of environment because, once more, the time scale is too short for that. The plausible reason for the observed shifts of e-nose signal are fluctuations of composition of ambient air, which apparently happen relatively quickly and, thus, are argued to be the major factor of the e-nose recognition instability, at least in the short-term.

### 3.2. Mid-Term Stability

In this subsection, we consider the e-nose performance for analyte recognition at the mid-term scale of few days to months. Such a time scale is the most usual one in practical studies described in literature [4,10,19]. During e-nose training at the mid-term duration, the whole assembly of short-term variations of its data is incorporated into the LDA model and they do not produce e-nose recognition instability anymore. The goal here is to estimate: (i) how long does it take for the training to encompass the elementary variances and reach the stable recognition at least for period of several days; and (ii) how long can the LDA model built in this way be used successfully. Concerning the second issue, we discuss the recognition of e-nose data obtained after a pause of about month following the successful initial training.

When we take into consideration a time scale of months, the season variations of ambient air come first into effect. Here we present the measurements which have been performed with the e-nose exposed under indoor conditions, in a laboratory room, but still are influenced with both changes of outdoor air and variations of minor indoor additive emissions in dependence primarily on the temperature inside the building.

For testing the e-nose, we have chosen in this study some practically important objects: meat of two staleness stages and honey of varying origin. Previously, we and other groups have shown that the meat quality could be well controlled by e-noses using synthetic/purified air [13,19,28,34], but the recognition of honey of different kinds is a more difficult task. It is shown that recognition of honey origin is usually possible in solution with the help of e-tongues [41,42], or under specially prepared carrier gas mixtures [43]. Unlike these methods, here we have employed the simple headspace sampling of our metal-oxide film array-based e-nose for both the objects, meat and honey, as the approach closest to the requirements of practice. It is worth noting that using ambient room air as a background for e-nose operation complicates significantly the analyte analysis when compared to data obtained in pure and stable filtered air as was done in [34]. Therefore, here we have chosen to discriminate meat at only two stages of staleness, the fresh one and the meat stored for 24 h at room temperature. The latter one yields an example of spoiled meat. The exposures of the e-nose to meat and honey have been performed in two measurement series: the first one has continued for 8 calendar days (6 measurement days) and the second one has been performed during 22 calendar days (18 measurement ones). Between the measurement series, the e-nose has been switched off and the multisensor array has been cooled down. The pause of six weeks between the series has been made in order to test the compatibility of e-nose data collected in both measurement series at separate times. The measurements of honey have been performed at two concurrent series, too. The first e-nose exposure series to honey have been carried out with samples harvested in the same florification area but at different collection times (origin: Kyrgyzstan, Sary-Chelek, harvesting time is June–July of 2016). The second e-nose exposure series to honey have been performed with samples stemming from two different harvesting regions (Sary-Chelek and Toktogul, Kyrgyzstan, where the harvesting time is July 2016). The honeys have been exposed to the e-nose being placed into Petri dishes. The same e-nose unit has been used for recognition both subjects, meat and honey, in all the measurements presented.

**Figure 3:**
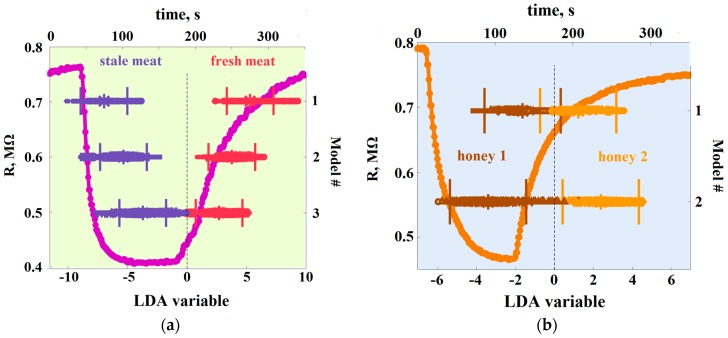
The results of LDA processing of the e-nose data to discriminate meat freshness (**a**) and honeys (**b**). The curves in the background show typical transitions of the resistance of exemplary sensor segment of the array during the exposures. The centers of LDA model classes are marked by asterisks symbols and the confidence intervals of 95% probability for each class are indicated with vertical bars. The meat freshness recognition models are numbered as: 1–model of the first series; 2–the second series one; 3–the combined model of both the series. The honey classes’ recognition models: 1–the samples of different harvesting times, 2–the samples of different harvesting places.

Typical evolutions of a sensor segment resistance over exposures to the both analytes are shown on the background plots of Figure 3a,b. The resistance goes down according to general chemiresistive effect observed in n-type SnO_2_–based sensors. Figure 3 shows also the one-dimensional LDA model intervals and corresponding E-nose training data obtained in the course of studying meat and honey.

In the case of meat freshness discrimination (Figure 3a), one can see that the LDA classes related to first series measurements have a between-center distance of about 12 units (LDA or Mahalanobis units) while these ones related to the second series measurements are distanced in 8 units. In terms of LDA discrimination, it means that the e-nose data obtained during the second series incorporate more data variations than these recorded at first series. The higher dispersion of the second series data cannot be caused by the drift of sensors of the e-nose array because the time duration of both series is still not sufficient for a significant drift (cf. Section 3.3). Figure 3b depicts the e-nose data regarding the discrimination of honeys in one-dimensional LDA space. One can see that the recognition of honeys of the same harvesting place is rather difficult because the confidence intervals of the corresponding analyte classes are overlapped. As in the case of meat freshness recognition, we would expect a higher separation level for the honey origin recognition in a purified air, too. The class separation of honeys of different origins in Figure 3b is higher: there is no class overlap. Therefore, the e-nose could recognize these analytes even in ambient air.

**Figure 4:**
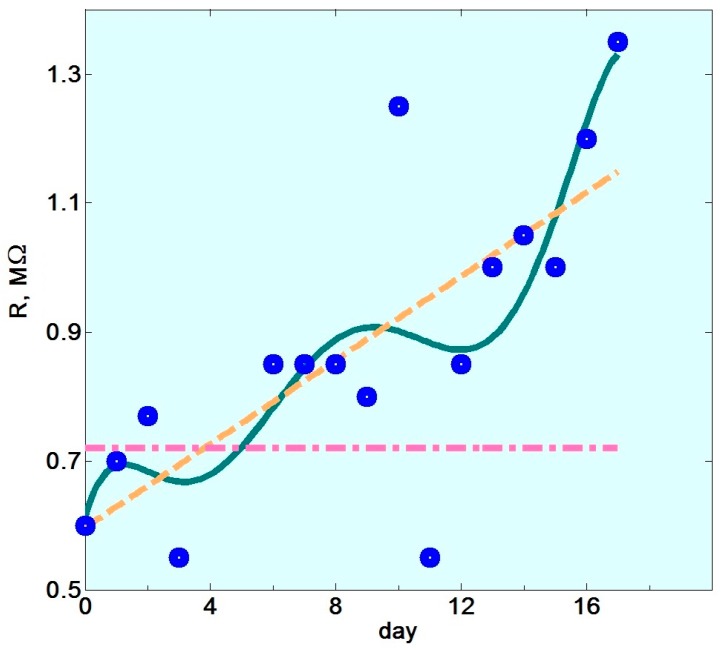
The time evolution of resistance of the exemplary sensor segment (#33) of the e-nose array measured in the mornings during the second measurement series (blue points) versus the day. Dashed yellow line indicates rather steep linear resistance trend. Solid green line discloses the step-like character of resistance curve under approximation by polynomial function of sixth degree. The dash-dotted red line marks the resistance level measured during the first series.

To approach the temporal change of e-nose signal directly, Figure 4 shows the evolution of a segment resistance of the array measured every day (in mornings) during the second measurements series. The linear approximation indeed indicates a steep increase in the mean resistance value whose rate would virtually contradict the results given in [16] and in Section 3.3. However, the more accurate polynomial approximation of the curve in the figure indicates the step-like nature of the resistance changes, which is not characteristic of drift. The physical mechanisms of oxygen diffusion [17] underlying the drift of gas sensor properties have a systematic character [16], so, the observed resistance “steps” reflect rather a purification of air by its cooling caused by abrupt weather changes during the autumn measurement period. This second series of e-nose measurements have been carried out during the middle of autumn season, including even few days with a snowfall.

Moreover, the sensor resistance jump from 0.8 MΩ to approx. 1.25 MΩ observed on the 9th day has been caused by opening the window in the lab that day. It further indicates that the impact of ventilation and corresponding changes in ambient air can be rather strong. In addition, we notice that during the first series of e-nose measurements when the summer weather has been quite stable, the resistance variations of sensor segments was much less pronounced; in the specific case of the same segment whose data are shown in Figure 4 these variations were within less than 5%. Neither drift nor abrupt changes of sensor resistances were observed during the first series period. Further confirmation of the absence of sensor drift during the period between the series is that the resistance level corresponding to the beginning of second series equals approximately to that of the first series end. On the other hand, the increased data variation during the second series of e-nose measurements cannot be reasoned with the assumption of sensor poisoning since the latter results in a decrease of oxygen content in the sensor oxide bulk or surface and the corresponding reduction of the sensor resistance. Contrary to that, Figure 4 demonstrates clearly increasing the sensor resistance value with time. Thus, the most reasonable explanation for the lower separation of LDA clusters observed in the second series of e-nose measurements (Figure 3a) is ambient air variations. Because the data of the second series have been collected over a longer and weather-turbulent time period, more variations are included. We have built the LDA clusters related to analytes taking training data from the both series; see model #3 in Figure 3a. In this case, the analyte-related clusters are located closer to each other with 6.5 units distance between the centers. It means that the both series data contribute variations of different kinds into the joint training pool. In this way, such a “joint” LDA model comprises more variations than every of the particular ones and therefore the separation of the classes is lower.

In order to clarify further the impact of variations of ambient air on the e-nose signal, we compared the data obtained in the current study to ones taken to analyze the meat freshness in the thoroughly purified air reported earlier in the [34]. For the reason of comparison, we have taken a similar sampling period of about a month. The distance between LDA clusters built in this case is in the range of 30–65 units that is much higher value when compared to the reported above distances observed while the non-purified room air has served as a background.

**Figure 5:**
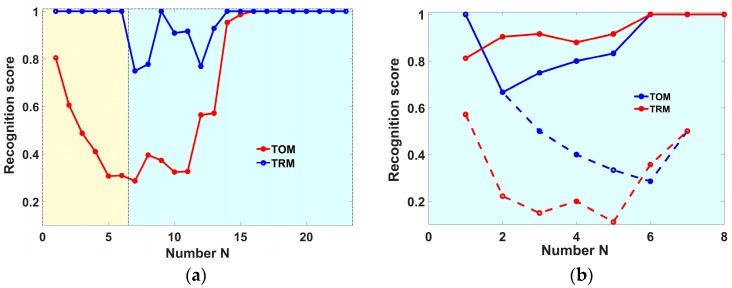
The *S_TOM_*(*N*) and *S_TRM_*(*N*) functions related to e-nose measurements concerning the recognition of meat freshness (**a**) and honey origin (**b**). *N* is number of days of e-nose measurements at the two series: (**a**) 1–6 are first series, 7–24 are the second series; (**b**) 1–7 are first series, 1–8 are the second series. At a point with the abscissa *N*, the training pool includes *N* days [34,36]. The *S_TOM_*(*N*) gives the LOO scores for this training pool. The *S_TRM_*(*N*) gives the recognition scores of all the involved E-nose measurements taken after the *N*th day.

The stabilization of e-nose recognition is further approached employing the *S_TRM_*(*N*) and *S_TOM_*(*N*) curves. In this subsection, the number *N* is the number of measurement days which data are included into the training pool. Every day includes a sequence of e-nose exposures. Both the meat measurement series have been performed with samples of the same meat sort and the same classes of freshness. Therefore, the data of two series can be included in the training pool one after another. The samples of two honey measurement series belong to different discrimination classes. They have been analyzed separately. Figure 5 shows the behavior of recognition scores in dependence on *N* for the meat (a) and honey (b) studies. The *S_TOM_*(*N*) curve at the plot 5(a) drops from 1.0 to a value of approx. 0.8 when the first measurement day of the second series is included into the training pool. On the contrary, S*_TRM_*(*N*) curve there starts going up just after the inclusion of e-nose data of the second measurement series into the LDA model. This behavior of the two recognition score curves clearly highlights the substantial differences between the e-nose data in two series. We attribute this difference again to deviations of air background rather than to other factors. As to the possible sensor drift, the 1.5 months interval between the two e-nose measurement series is not sufficient for a significant drift of the electrophysical properties of the metal oxide sensors (see [16] and the results of Section 3.3). Another reason is that the sensor array which we employed in these measurements has been rather aged prior the study at the operating temperature for many weeks. Moreover, the array has been stored at room temperature during the pause between series: as it is well known, the lower temperatures reduce the evolutionary dynamics of electrical properties of metal oxides [17].

Figure 5b draws the *S_TOM_*(*N*) and *S_TRM_*(*N*) curves calculated for the e-nose signals recorded under exposure to the honey in the two measurement series. The dashed curves relate to the recognition of the honeys harvested on the same place but at different times (model #1 in Figure 3b). The solid curves represent the discrimination of honeys stemming from two different regions (model #2 in Figure 3b). For this latter case, when number of measurements *N* to include into LDA model is enhanced, the curves gradually reach the 100% recognition score. This corroborates the conclusion made while considering Figure 3b that the odors of honeys of different regions differ substantially. On the contrary, the e-nose response to the odors of honey from the same region but harvested at different times do not differ sufficiently; their recognition score approaches 50% that means no recognition is possible, at least in ambient air. However, there is a possibility that the initial *S_TOM_* level could stay high if the background gas remains stable enough. The observed drop of *R_TOM_*(*N*) curve yields an example where the e-nose cannot properly recognize the analytes if they are of lower difference when compared to stronger variations introduced by changing conditions. In this case, there is no way to provide the proper e-nose operation except for taking a stable background gas which would have low variations of similar or less magnitude when compared to ones made by differences of analytes.

The behavior of *S*(*N*) curves drawn in Figure 5a supports the introduction of add-training procedure. The addition of a few e-nose measurements of the second series into the training pool collected at first series allows the e-nose to get 100% recognition of the following exposures to analyte in spite of the apparent differences between the two series conditions. However, it is a matter of further investigation and justification how many add-training measurements are needed to update the LDA model and how frequently should such a recognition model be updated in order to maintain the e-nose recognition level in different practical applications.

### 3.3. Long-Term Stability

**Figure 6:**
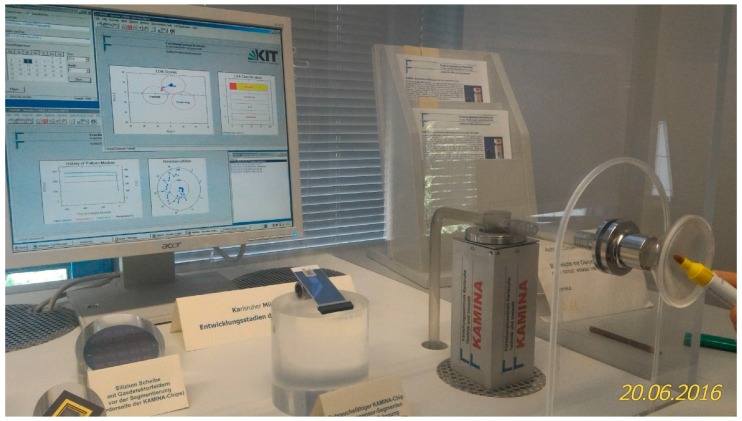
The photo of the e-nose installed in Karlsruhe Institute of Technology to demonstrate the performance of analyte recognition. Next to the e-Nose, one can see a mounted multisensor array chips and the sputter mask to fabricate it. The analytes to recognize are the odor emitted by felt pen and fuel gas emitted by lighter. The photo has been taken when the e-nose is exposed to the pen.

In this subsection, we address the long-term stability of the e-nose operation observed in a period of years. We will account for data obtained with a facility, which has been constructed in 2002 at Karlsruhe Institute of Technology in order to demonstrate the long-term e-nose performance for analyte recognition. Figure 6 shows the photograph of the facility under operation.

The e-nose interfaces to a PC equipped with custom-made software which reads out and stores the signals from the chemiresistive multisensor array to the computer. Since 2008, the e-nose has worked continuously with the same multisensor array chip operated under heating to a maximum of 330 °C. Only a couple of times, the chip has been cooled down for external technical reasons. Since 12 January 2009, the facility is situated in an ordinary room whose door has been left open to maintain a good air contact with the staircase hall of the building. Since 11 January 2012, the door of the room has been kept mostly closed being opened only from time to time to allow making e-nose measurements versus analytes that limit the contact with the outside air. Since April of 2009, i.e., already for more than 8 years, this instrument operates under LDA processing to distinguish the odors emitted from a felt pen and fuel gas delivered by a lighter while the ambient air is used as the background gas. The sampling interval to read out the multisensor array resistances is about 1 s; the value of median sensor array resistance is saved every 30 s to PC.

**Figure 7:**
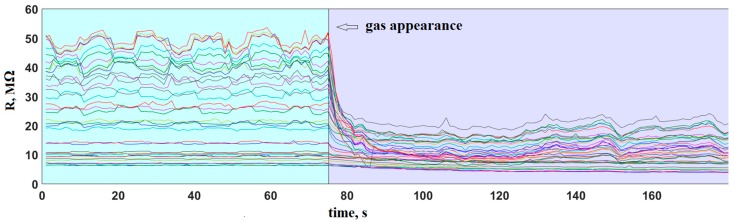
A typical *R*(*t*) behavior of the array segments in the e-nose during an event of its exposure to fuel gas emitted by a lighter. The resistance curves of different segments are marked with different colors. The arrow points the moment of gas appearance.

The add-training procedure has been implemented into the e-nose in combination with the LDA recognition method for long-term measurements as the in-built software. After an intensive rate of analyte probe exposures to the e-nose at the beginning of the operation period, the further exposures have occurred rather sporadically. As the consequence, the obtained data are not so systematic. However, the e-nose signal at ambient air has been continuously recorded and temporarily stored in the PC. The appearance of an analyte probe (called “event”, hereafter) has been automatically detected by the in-built software via a sufficient quick drop of the median sensor array signal. The typical behavior of sensor segment resistances of the e-nose following an exposure to fuel gas from a lighter is illustrated in Figure 7.

The e-nose software continuously retains the last 200 consecutively measured multisensor array signal points. These recent points enable alarming by the appearance of events and a posteriori referencing to the directly preceding air state data. In this way, the raw multisensor array data are pre-processed prior feeding to LDA in two steps. First, the sensor segment resistances *R_i_* are normalized by referencing to the corresponding time-averaged resistances recorded in the preceding air $R_{i}^{air}$. At this stage of preprocessing, the resistance profile over the array is segment-wise divided by the reference resistance of corresponding array segment as follows:

(1) $$R_{i}^{ref}=R_{i}/R_{i}^{air}$$

where *i* is the segment number in the multisensor array. Second, the obtained referenced profiles $R_{i}^{ref}$ are divided then by their median value $R_{med}^{air}$ taken over the array:

(2) $$R_{i}^{ref}\to R_{i}^{ref}/R_{med}^{air}$$

These pre-processed array signals are recognized by the current LDA model and, if accepted, are added to the training pool in order to correct the LDA model. Each e-nose data point is classified automatically as described below to belong to one of the target analytes or as “other”. The LDA clusters of the target analytes are built with radius corresponding to 0.95 confidence level, multiplied by a factor of 5. This factor is introduced to enlarge the radiuses in order to secure the analyte recognition by e-nose if the multisensor array data are moderately scattered.

**Figure 8:**
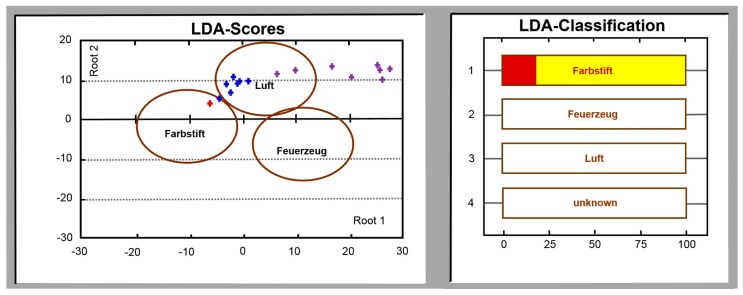
The PC screened image of the e-nose software under the long-term operation. Several last measurement points are displayed with the blue crosses while the last point is marked by the red color. Blue crosses and the classification table at the right side show a transition of e-nose state from the “ambient air” (“Luft”) to the “odor induced by felt-pen” (“Farbstift”). The violet crosses indicate e-nose signals which are classified as “others” (“unknown”). The red bar fraction shown in the right plot corresponds linearly to the position of red cross: it disappears when the cross is on the ellipse’s boundary and takes the complete bar, if the cross is in the center. The class “lighter” is marked as “Feuerzeug”.

It is worth noting that the strong difference of the test analyte odors here results in sufficient LDA interclass distances for such an enlargement. If an event results in that the e-nose signal appears in the LDA space outside all the class clusters related to the target analytes, it is qualified as unknown and is marked as “other”. When the e-nose signal falls into the LDA class related to the ambient air, while the multisensor array median signal changes rather significantly, this event is qualified as “error”. The image of the PC display provided by the e-nose software is shown in Figure 8. Normally, the indication of “others” and “error” events appears at e-nose exposures to occasional odors which do not belong to the target analytes.

The e-nose data recorded at every analyte exposure that is classified as the “pen” or “lighter” are taken into the training pool together with the data measured at preceding ambient air to update the LDA model. Then, the obsolete data are removed from the training pool and the LDA model is updated. The whole procedure is performed instantly and completely automatically. The obsoleteness of e-nose data is judged here by their number in the training pool: each LDA class related to test analytes except for the background air has to collect data of last six properly classified events. Accordingly, the LDA class related to background air is based on the 12 corresponding events. All the e-nose event data and the LDA model parameters are constantly saved. The initial e-nose training has been carried out by an operator while the further actions are automatic ones as described.

During the 8 years period of the e-nose operation under the described mode no misrecognition of the two analytes has been registered. Although we cannot exclude the possibility that some e-nose exposures to the odor from the pen or the lighter have been qualified as “others” or “errors”, such qualifications are not considered as misrecognitions. We have observed in practice that the LDA-transformation matrix has changed constantly during this long-term e-nose operation due to the instability factors. Nevertheless, it has still preserved the characteristic features related to the analytes and maintained the high separation of the corresponding classes. It is worth noting that because the add-training procedure works automatically, no e-nose recalibration procedures performed by an operator are necessary. It means no additional expenses are required. The success of the analyte recognition by the e-nose is fully dependent on the distances between the classes in the LDA space. In practice, when the LDA classes related to different analytes are quite close to each other, like ones described in Section 3.2 (cf. the analyte class distances in Figure 3 and Figure 8), the misrecognitions are to be expected and the operator’s supervision can be necessary. In addition, all the parameters of add-training procedure have to be adjusted to a particular application.

**Figure 9:**
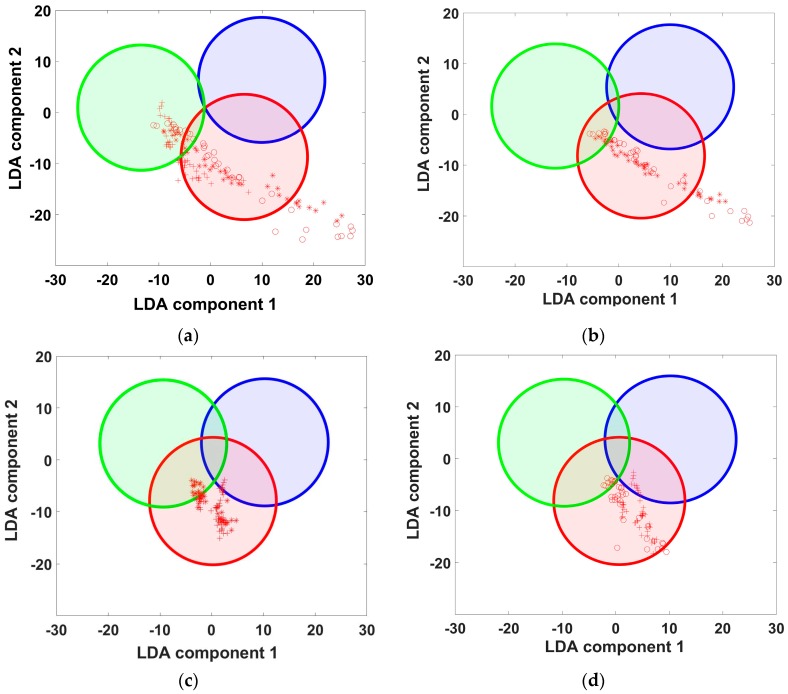
LDA diagrams illustrating e-nose recognition of three deviating exposures to analytes. (**a**)-recognition with the LDA model built on the training pool which contains data of 35 previous exposures: the recognition score is 0.48. Inclusion of only one of the deviating e-nose data into the training pool of the LDA model radically improves the recognition of the two other deviating exposures: (**b**–**d**) show the three possible variants of the inclusions; the scores are 0.8 in (**b**) and 1.0 in (**c**,**d**).

In this connection, we would like to address the most significant question of the add-training procedure: are the expenses of the add-training of a similar magnitude as the expenses of an e-nose re-training at the current time point? For example, we may take the e-nose data collected for the period of 5 months between April 2009 and September 2009. This dataset comprises 35 e-nose exposures to analytes, which we use here as the training pool (contrary to the real-life e-nose operation with six samples of each analyte in the training pool). Following this training, the next e-nose exposure has occurred to the lighter after the 6-month pause on 12 March 2010 with three exposures on the same day. These exposures have resulted in much deeper drops of the sensor resistances than the previous ones. Rather probably, the deeper drops have been caused by higher analyte concentrations and possibly some additional gas components emitted from the new lighter exposed. Using the training dataset stored in the e-nose memory from the data obtained in 2009, the LDA model would poorly recognize these three e-nose exposures; the mean score of the correctly recognized measurement points is 0.48. However, the inclusion of data of just one of these three exposures into the training pool sufficiently changes the situation as shown in Figure 9.

As one can see, this addition allows the software to recognize perfectly the data of the other two deviating e-nose exposures, although the data of only one exposure has been added versus the huge dataset stored in the LDA training pool. Thus, this example supports the supposition that add-training procedure is not expensive: it needs only few exposures to adjust the training dataset to new conditions in order to update the LDA model for maintaining proper analyte recognition.

**Figure 10:**
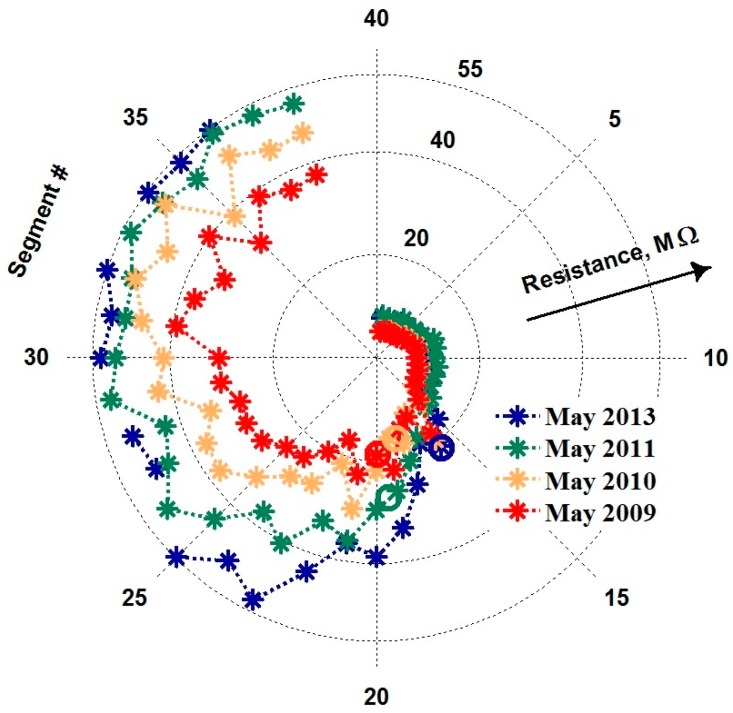
Distributions of the segment resistance in the e-nose array (cf. [23]) recorded under ambient air during the period of four years, 2009–2013. Since the maximum measurable resistance of the sensors in the array of the e-nose is 55 MΩ, not all the sensors are plotted here. The positions of the median values taken over the array are encircled. The profiles are chosen to correspond to the similar season (spring).

Now let us return to fundamental causes of e-nose instability. The multisensor array chip employed in the e-nose for the long-term operation has been in use for many days prior to the reported long-terms measurements under the add-training procedure. Therefore, the primary short-term drift of the sensor resistance is not observed in the saved data. However, the mid-term drift of the sensor resistance, which is often related to a diffusion of vacancies in metal oxides [17,44,45], has been ascertained as shown for example in Figure 10 (It is worth noting that the cogged character of the sensor array profile is not essential to be accounted for in the present study though it is rather characteristic for the metal oxide multisensor array chips. It appears during a long e-nose operation period, due to oxygen ion migration [45,46]).

**Figure 11:**
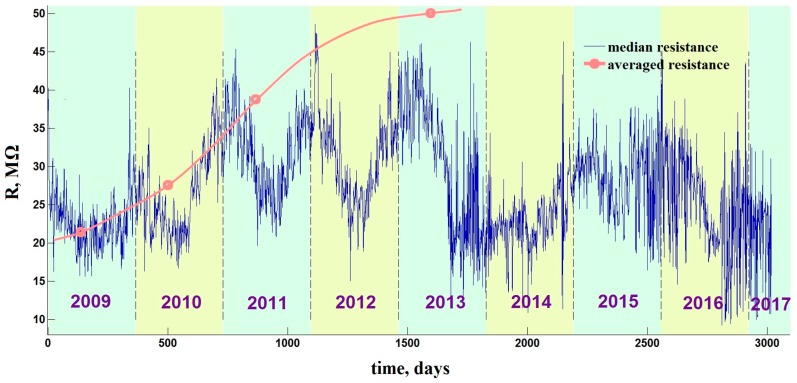
The long-term evolution of median sensor resistance of the array in the e-nose measured from 2009–2017. The median evolution expresses the year-season and short-term variations. The averaged resistance represents the mean value of few typical sensor segments #22–25 from the array; points correspond to the distributions given in Figure 10 to be fit by curve built by their polynomial approximation. The averaged points illustrate the long-term drift and the eventual stabilization of sensor resistances. Oscillations of the curve to be seen since the fourth quarter of 2015 are reasoned by jumping of the position of median from one segment to another.

The drift has almost disappeared after 4 years of the e-nose operation: the resistance values of sensor segments reached a maximum to the end of 2013 and remained approximately constant until 2017. That means that under the stable heating conditions at temperature about 330 °C the chemiresistive metal oxide material has reached a thermodynamic equilibrium with the environment and become rather stable. However, at the preceding period of e-nose operation between 2009 and 2013, the sensor resistances drift to upper values as clearly seen in Figure 10 and Figure 11. Nevertheless, it must be emphasized that while the sensor resistances have been drifting, the e-nose operated under the add-training procedure has continued to recognize the analytes without errors. We can note that these data do not support the conclusions reported in [9], which argues that no application of e-nose is possible if the sensors are instable. Thus, the applicability requirements on the e-nose have to be revised.

**Figure 12:**
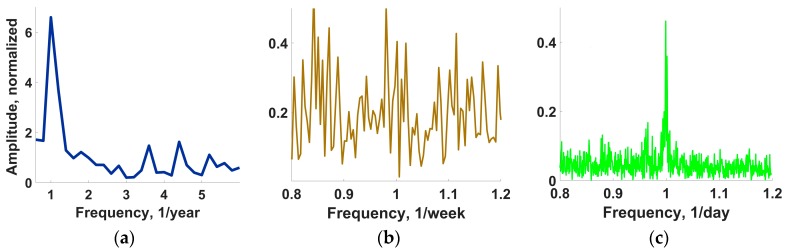
The Fourier spectrum of the median resistance value of the multisensor array in the e-nose measured in the years 2009–2017. The spectrum sections are given near the (**a**) annual peak; (**b**) weekly peak; and (**c**) daily peak. Yearly and daily peaks exceed essentially the local mean-spectrum level; the weekly peak does not.

Another major factor of e-nose instability, which we consider here as the most significant one, is the background air variation. Figure 11 draws the long-term behavior of median sensor resistance value over the multisensor array of e-nose recorded for more than 8 years (2009–2017). One can see the essential short-term fluctuation spikes of the median value. It is important to note that these fluctuations in the median resistance value do not disturb the analyte recognition by the e-nose, which is 100% under the add-training approach. Moreover, the year oscillations of the median resistance curve are even more expressed ones reflecting the seasonal environmental variations. The annual and daily peaks are particularly pronounced in the spectrum of the median curve as we show in Figure 12, which gives three frequency intervals of the Fourier spectrum of the long-term median resistance function shown in Figure 11.

The sections are taken in vicinity of the annual, weekly and daily frequencies. It indicates that daily and annual variations dominate over other temporal variations of the sensor resistance values in the multisensor array. However, the annual variations are definitely predominant when taken in the absolute value, so the seasonal data variations are most important, which further support the results of Section 3.2 for necessity of adopting these variations by the add-training.

**Figure 13:**
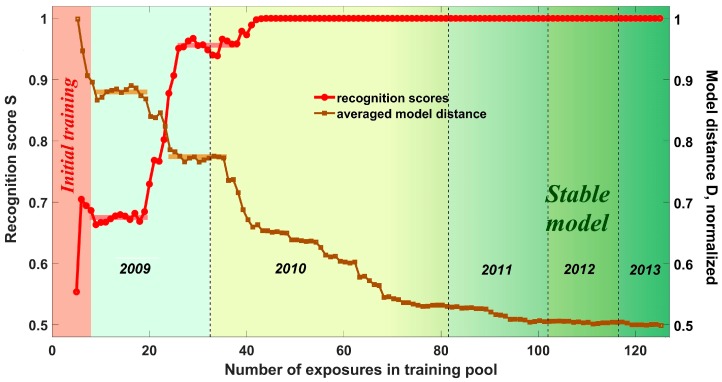
Evolution of *S_TRM_*(*N*) and the characteristic distance of the analyte-related clusters in the LDA space, *D*, with increasing the number *N* of e-nose exposures included into the training pool. The data of e-nose exposures to odors of pen and lighter have been recorded between April 2009 and September 2013. The distance *D* is normalized by the value observed at the initial e-nose training.

The demonstrated proper performance of the add-training procedure in the long-term operation of the e-nose still leaves open the question whether the add-training is necessary for this facility at all. Indeed, it cannot be ruled out a priori that the analytes are so different for the e-nose that the initially trained LDA model would recognize successfully the analytes in the same way during all the following years. As the “initial LDA model”, we consider a model obtained via a training of the e-nose over several days until it starts to recognize the test analytes properly. Such models are produced in almost every published research in e-nose application (e.g., [10,12,19,20]). The initial LDA model of the e-nose has been obtained in this study just after approx. seven measurements. Then the e-nose has been left to be operated by the automatic add-training procedure. Figure 13 shows the *S_TRM_*(*N*) curve for the complete e-nose data recorded between April 2009 and September 2013; *N* enumerates here the exposures included into the training pool. We have to mention that *S_TOM_*(*N*) curve stays for these data always at the 100% score level as well as the real-life add-training recognition.

The *S_TRM_*(*N*) curve primarily aims to estimate whether the initial LDA model is able to recognize all the following data recorded for several years. Here, the number of e-nose exposures which data has to be included into the training pool differs from year to year. Contrary to the add-training procedure, the TRM one does not exclude the older data from the pool. In this way, the *S_TRM_*(*N*) curve grows constantly in Figure 13 although there are two plateaus near the e-nose exposures with numbers *N* = 7–20 and *N* = 25–38. During the second quarter of 2010, the *S_TRM_*(*N*) value reaches 100% and remains at this level during the following growth of the training pool. Another curve, *D*(*N*), drawn in Figure 13 relates to the evolution of the position of cluster centers related to analytes in the LDA space with addition of the measurements into the training pool. The characteristic distance *D* here is the mean Euclidian distance between the class centers and the center of the LDA coordinate system. This distance serves generally as a measure of class separation by LDA. As one can see, the *D*(*N*) curve behaves oppositely to the *S_TRM_*(*N*) one. Such a behavior agrees to the known property of inter-class Mahalanobis distances: an addition of measurements reduces the inter-class distances as far as new data features are introduced into the training pool and the additional variances inflate the class clusters (cf. e.g., [34]). Accordingly, as the LDA distances are related to the class radii, the values of *D* are reduced. As a result of this ongoing addition of new data into the training pool, the LDA clusters related to analytes encompass ever more variants of possible analyte odor manifestations and the recognition is enhanced (this is an ordinary behavior for well-separated analyte-related clusters in the LDA space as in the case of the long-term e-nose study discussed here. However, it is worth noting that in a case the clusters are not sufficiently separated in the LDA space, their expansion under adding new measurements can result in more expressed overlap and a reduction of the recognition scores (cf. Figure 5b, dashed curves)). The appearance of plateaus in the *D*(*N*) curve of Figure 13 indicates that no additional features are added to the LDA classes during these periods and the new incoming e-nose measurements contain the same analyte-specific information already stored in the training pool. Obviously, because the positions of analyte-related clusters in LDA space do not change during the plateau periods, the recognition score stays stable, too. Following the evolution of the *D*(*N*) curve one sees that the position of clusters in the LDA space is sufficiently stabilized around 2011. The *S_TRM_*(*N*) score has been stabilized earlier, in February 2010, because the e-nose signals at later analyte exposures match the corresponding Voronoi sectors [38] in the LDA space even despite that the centers of the analyte–related clusters still shift.

The data shown in Figure 13 mean that the analyte-specific features of e-nose signals related to background air and odors of pen and lighter are already accounted for by the training pool at the end of 2011 at the latest, because the distances of LDA model do not change anymore after this date. At the same time, Figure 11 and Figure 12 evince that the multisensor array resistances change after the end of 2011 due to the sensor aging drift. This contradiction can be explained as follows. The drift changes of the sensor segment resistances in the array are systematic, i.e., are monotonous and similar for all the sensors in the e-nose array. Therefore, the particular features related to such a drift are accounted by LDA model at a primary stage, before the end of 2011, and are automatically filtered out by LDA later on (The differences of sensor segment resistances, not the values themselves, contribute to the LDA transform coefficients. The employment of the differences of values disregards the drift since it is similar for all the sensor segments). The only inherently unpredictable variations of the e-nose signals due to ambient air composition fluctuations are continuously considered by LDA to have the effect on the recognition models. These variations are responsible for the long-term modifications in the LDA model parameters up to 2011, but even they are encompassed after that time.

The noticed air variations have different time scales, which can be distinguished by the plateaus appeared at the *D*(*N*) curve in Figure 13 at approx. *N* = 7–20 and *N* = 24–36. The scale that could be primarily considered is the daily variations (cf. the daily peak of the median sensor resistance spectrum in Figure 12c). As one can see in Figure 13, the corresponding variations of the e-nose signal are already accounted by LDA following 7–10 measurements contained in the training pool. The appearance of first plateau just around this number indicates a completion of the initial training. It corresponds well to the initial e-nose training discussed in the Section 3.2. Such a stage of training pool completeness could be observed in published application-targeted studies of e-noses (see, e.g., [4,10,11,12,19,34]). The LDA model parameters obtained at this plateau region do not change significantly, too. Then, the season variations come into play. The first set of season variations is accounted by LDA to some extent by the end of 2009 at the *N* ≈ 25 when the second plateau appears. The changes of background air under winter conditions, which have introduced more peculiarities to vary the e-nose signal soon after beginning of 2010 at the *N* ≈ 35, result in the abrupt change of *D* value, so the data of Figure 13 make it possible to conclude that: (1) the sensor drift in the e-nose is automatically compensated by the LDA model; (2) the initial training is not sufficient for the e-nose to operate over the long-term and add-training is necessary.

Here, we would like to examine once more the e-nose requirements which must be met in order to employ this device in real-world applications. According to [9] the necessary requirements are periodicity, dissimilarity and stability. The periodicity of analyte classes, as it is described, obviously results in their high dissimilarity after application of any transform, which transfers the original feature space into one with maximized distances between the class clusters as LDA does. Therefore, the periodicity should relate to dissimilarity and has to be removed from the list of e-nose requirements. Moreover, we see that regular instabilities such as the sensor aging drift in the e-nose array can also be easily coped with by pattern discrimination/recognition algorithms like LDA. Even the irregular but temporal instabilities such as the air composition variations can be dealt with using a proper calibration procedure like the add-training being discussed here. In this way, the in-class dispersion of data is no longer determined by all the possible variations recorded for a total e-nose measurement period but it is caused by only current variation diversity. Such rather short-term variations could be easily accounted for by application of the described add-training procedure. Correspondingly, the dispersion of data within the classes is much less and the dissimilarity of classes is much higher. Thus, the instabilities both of the sensor drift and of the air composition variations do not prevent e-noses to operate in long-term real-life applications. Finally, we find that only the dissimilarity, as it is formulated in [9], is the key feature to consider whether the device fits to the task. A high dissimilarity for e-nose signals is still required, which means in fact that the distance between the analyte classes in the transformed coordinate system generated by pattern recognition algorithm is rather discernable against the background of the “noise” caused by variances within the classes. Thus, the dissimilarity requirement on e-noses is just a version of the well-known signal processing theory rule (cf. [47]): “If, and only if, a signal is discernable over the background of random noise it can be recognized”. Other requirements are superfluous.

## 4. Conclusions and Outlook

In view of the main topics of the present study, we first summarize that the ambient air variations seem to be the most significant factor to disturb the stability of recognition by the e-nose based on metal-oxide multisensor arrays. This is supported by our data recorded over different time scales of e-nose operation ranging from tens of minutes to several years in an ambient indoor air background. The sensor aging appears to have less influence on the e-nose performance with time because it can be overcome with the help of relatively simple automatic training procedure. The third important factor, sensor poisoning, has not been observed in this study.

The application of an add-training procedure allowed us to demonstrate for the first time an e-nose which continuously executes a recognition task for many years. This procedure has provided adjusting the LDA recognition model and has stabilized the analyte recognition by e-nose within all the time scales, including long-term operation. We have observed a 100% recognition of analytes just after the initial training of the device for few days over the total following period of its operation in the case of pronouncedly distinct analyte additives in the ambient air. Thus, the e-nose can operate quite stably and reliably for a very long period. Although the obtained results relate to an e-nose based on metal-oxide sensor elements, it is reasonable to suggest that they could be extended to other e-noses of different kinds since the accented influence of the ambient air variations affects various sensor array types.

One more remark could be addressed to such a point that the long-term stable analyte recognition by our e-nose has been achieved versus quite distinct analytes that is not characteristic for great majority of practical applications. Therefore, the next step is required to prove the efficiency of add-training procedure in long-term industrially-orientated tasks.
