# Erratum: Kiselev, I., et al. On the Temporal Stability of Analyte Recognition with an E-Nose Based on a Metal Oxide Sensor Array in Practical Applications. *Sensors* 2018, *18*, 550

**DOI:** 10.3390/s19163525

**Published:** 2019-08-12

**Authors:** Ilia Kiselev, Victor Sysoev, Igor Kaikov, Ilona Koronczi, Ruslan Adil Akai Tegin, Jamila Smanalieva, Martin Sommer, Coskan Ilicali, Michael Hauptmannl

**Affiliations:** 1 Breitmeier Messtechnik GmbH, Englerstr. 27, 76275 Ettlingen, Germany; 2 Laboratory of Sensors and Microsystems, Yuri Gagarin State Technical University of Saratov, 77 Polytechnicheskaya str., 410054 Saratov, Russia; 3 National University of Science and Technology MISiS, 4 Leninskiy pr., 119991 Moscow, Russia; 4 Institute of Microstructure Technology, Karlsruhe Institute of Technology, Hermann-von-Helmholtz-Platz 1, 76344 Eggenstein-Leopoldshafen, Germany; 5 Science and Technology of Nanosystems, Karlsruhe Institute of Technology, Hermann-von-Helmholtz-Platz 1, 76344 Eggenstein-Leopoldshafen, Germany; 6 Faculty of Engineering, Kyrgyz-Turkish Manas University, Mira Avenue 56, 720044 Bishkek, Kyrgyz Republic

The authors wish to make the following corrections to this paper [1]:

The labels of curves in Figure 5a are in wrong order. Please replace the following: 
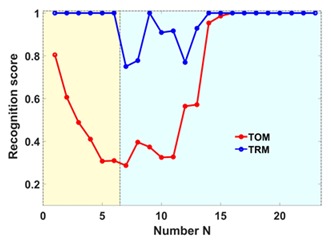
 with 
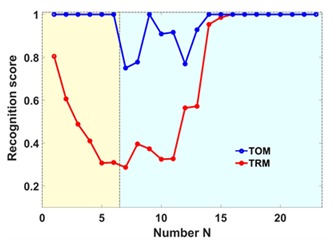


The authors would like to apologize for any inconvenience caused to the readers by this unintentional mistake.
